# Title: Citric Acid-Stabilized Amorphous CaCO_3_ as a High-Surface-Area Carrier for Functional Food Ingredients

**DOI:** 10.1021/acsomega.6c02212

**Published:** 2026-07-20

**Authors:** Daichi Sakawaki, Khairunnisa Mohd Paad, Rena Nagaoka, Yasuteru Mawatari, Mayumi Ikeda-Imafuku, Tatsuya Fukuta, Prakasan Nisha, Kazunori Kadota, Ayaka Sawada, Shinya Yamanaka

**Affiliations:** † Division of Applied Sciences, 13317 Muroran Institute of Technology, Mizumoto-cho 27-1, Muroran 050-8585, Japan; ‡ Department of Physical Pharmaceutics, School of Pharmaceutical Sciences, 13145 Wakayama Medical University, 25-1 Shichibancho, Wakayama 640-8156, Japan; § Agro and Food Processing Technology Division, CSIR-National Institute for Interdisciplinary Science and Technology (CSIR-NIIST), Thiruvananthapuram 695019, Kerala, India

## Abstract

Amorphous CaCO_3_ (ACC) has an intrinsically large specific surface area, rendering it a promising platform for developing various functional materials. However, its practical use is severely limited by an extremely short lifespan, which is typically only a few seconds, before its spontaneous recrystallization into vaterite or calcite. In this study, we investigate the use of citric acid to suppress the rapid crystallization of ACC in a mixed ethanol/ethylene glycol medium and to obtain reproducible high-surface-area ACC under controlled conditions. Experiments involving systematic variations in citric acid concentration (0.00–1.33% w/v) revealed that crystallization was completely inhibited at concentrations above 0.39% w/v, as confirmed by X-ray diffraction. Brunauer–Emmett–Teller analysis showed that ACC samples synthesized with 0.44–1.11% w/v citric acid achieved exceptionally high surface areas (600–700 m^2^/g). The observed suppression of crystallization is discussed in relation to previously reported interactions between citrate species and calcium carbonate phases, rather than being directly demonstrated at the molecular level. As a proof of concept, curcumin was loaded onto the prepared ACC samples, and enhanced dissolution behavior was observed, which plateaued above a specific surface area threshold. This finding highlights the importance of the surface-area-to-cargo ratio in future formulation strategies. The resulting stable high-surface-area ACC is a promising platform for the design of functional materials.

## Graphical abstract

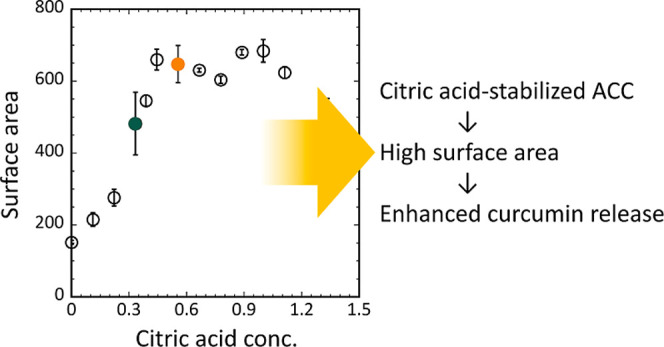

## Introduction

CaCO_3_ is one of the most abundant inorganic materials on Earth and a representative biomineral found in seashells, pearls, and crustacean exoskeletons.^1,2^ It is produced through biomineralization, a highly regulated process in living organisms. In recent years, the mechanisms underlying mineral formation have become an important research focus because the insights gained from the relevant studies can enable the rational design of new functional materials through controlled synthesis strategies.^3–6^ CaCO_3_ exists in several polymorphs, including calcite, aragonite, and vaterite.^1,7,8^ These polymorphs can be selectively formed by tuning various physical and chemical parameters (e.g., temperature, pH) and incorporating additives during synthesis, rendering CaCO_3_ a widely used model system in crystallization research.^9–12^ Industrially, the carbonation process affords high-purity CaCO_3_ with controlled particle size and morphology, but its specific surface area is typically ∼10 m^2^/g,^13^ limiting use where high loading and surface reactivity are required.^14^

Amorphous CaCO_3_ (ACC) has attracted considerable attention because of its inherently large specific surface area. However, ACC is extremely unstable, with a lifespan of only a few seconds before it transforms into its crystalline polymorphs.^14^ This rapid transformation has led to challenges in studying the formation of ACC and exploiting its unique properties for material applications. Although various additives such as carboxylic and amino acids have been used to inhibit crystallization,^15,16^ research specifically addressing the acid-assisted stabilization of ACC remains limited. Previous studies have shown that citric acid can alter the structure of ACC, prolong its lifetime, and modify its crystallization pathway.^14,17^ Despite this mechanistic progress, the translation to application-oriented studies remains limited, largely due to the lack of a reproducible stabilized phase and limited proof of function.

Building on these mechanistic insights, we hypothesize that defining a citrate concentration “stabilization window” can yield amorphous ACC with reproducibly high surface areas (>500 m^2^/g), and that these tunable properties will directly influence the loading and dissolution behavior of hydrophobic functional ingredients. In this framework, stabilized ACC is not only a structural curiosity but a potentially scalable platform material whose performance can be quantitatively mapped to its surface characteristics.

In this study, we target this application gap by demonstrating citric-acid-mediated suppression of ACC nucleation and inhibition of polymorphic transformation in dispersions. Citric acid was selected for its multidentate carboxyl groups and established safety as a food additive. Stabilized, high-surface-area ACC is particularly promising as a carrier for poorly water-soluble functional ingredients. As a proof of concept, we focus on curcumin—a hydrophobic polyphenol with antioxidant and anti-inflammatory activities that suffers from extremely low aqueous solubility—thereby motivating improved delivery strategies.^18,19^ Given that CaCO_3_ is an approved food additive and a common dietary calcium source,^20^ citric-acid-stabilized ACC represents a safe, ingestible platform. By establishing stabilization and demonstrating controlled curcumin loading/release, we lay a foundation for ACC-based carriers for functional food ingredients.

Moreover, by explicitly linking ACC stabilization, surface-area tuning, and release behavior, this work establishes an application-oriented framework that enables rational design of ACC-based functional materials. This approach provides clear performance metrics—such as a surface-area threshold for dissolution enhancement—that can be generalized to a broader range of hydrophobic nutraceuticals and bioactives. While citrate-assisted stabilization of amorphous calcium carbonate itself has been reported in previous mechanistic studies, the present work addresses a distinct application-oriented question. Specifically, this study establishes a reproducible citrate concentration window that enables the preparation of ACC with extremely high specific surface areas (>500–700 m^2^/g), and quantitatively links this structural parameter to the dissolution behavior of a hydrophobic functional ingredient. By explicitly correlating stabilization, surface-area enhancement, and release performance, the present study provides a framework for rational design of food-grade ACC-based carrier systems, rather than aiming to introduce a new stabilizing chemistry.

## Methodology

### Synthesis of ACC

Ethanol (99.5%, Kanto Chemical, Japan), ethylene glycol (99.5%, Kanto Chemical), and CaCO_3_ (99.5%, Kanto Chemical) were used without further purification to synthesize ACC. CaO was obtained by calcining 15.0 g of CaCO_3_ on a crucible in an electric furnace (FO 200, Yamato Scientific, Japan) at 1000 °C for 2 h.

A stable ACC dispersion was prepared by adding 6.4 g of CaO to 475 g of a mixed solvent of ethanol + ethylene glycol (7:3, w/w) inside a reaction vessel and stirring at 400 rpm.^21^ An ethanol/ethylene glycol mixed solvent was employed to suppress water-induced dissolution and recrystallization of ACC during the carbonation and aging processes. Previous studies by our group have demonstrated that reducing effective water activity in such organic solvent systems kinetically retards polymorphic transformation of calcium carbonate and enables retention of ACC-derived nanostructures.^21,22^

Carbonation was initiated in a mixture of CO_2_ and (flow rate, 0.3 L/min) and N_2_ gas (flow rate, 0.7 L/min). The reaction was terminated when the solution pH reached 8.5. The suspension was centrifuged at 3542*g* for 20 min to remove unreacted CaO, and the supernatant was collected for further analysis.

### Preparation of Citric Acid-Stabilized ACC

ACC samples stabilized with 0.00–1.44% (w/v, defined with respect to the 90 mL ACC dispersion prior to mixing) citric acid were synthesized. Briefly, the required amount of citric acid was dissolved in 20 mL of ethanol and 90 mL of the ACC dispersion. The mixture was then stirred (RSH-IDN, AS One, Japan) at 20 °C and 400 rpm for 1 h. When citric acid was added to the ACC dispersion, the ACC mixture turned cloudy. It should be noted that the observed “cloudiness” does not indicate macroscopic precipitation but rather reflects a change in the dispersion state of ACC particles. This behavior is attributed to alterations in colloidal stability and interfacial interactions induced by the addition of citric acid. After stirring, the ACC–citric acid mixture was aged at 60 °C for 24 h inside an oven to remove moisture from the ACC particles, washed with ethanol,^23^ and centrifuged at 6000 rpm (Micro Refrigerated Centrifuge 3700, Kubota, Japan) and 20 °C for 15 min. Ethanol washing was repeated three times to remove the ethylene glycol from the surface of the ACC particles. Subsequently, the obtained samples were dried in a vacuum dryer (AVO-200NS, AS One) for 24 h, and the solid particles were collected.

### Loading of Curcumin on ACC

Curcumin (99.5%, Kanto Chemical) was loaded onto reagent-grade CaCO_3_ and three types of ACC labeled ACC-Cur0.22, ACC-Cur0.39, and ACC-Cur0.56 (e.g., ACC-Cur0.22 to refer to the sample prepared with 0.22% w/v citric acid). Curcumin loading onto ACC was performed using a solvent evaporation method. Curcumin (300 mg) was first dissolved in ethanol (30 mL), after which 1.0 g of ACC was added, and the mixture was stirred under magnetic agitation for 30 min to ensure homogeneous adsorption.

The solvent was subsequently removed using a rotary evaporator (REV202M, Yamato Scientific) at 80 °C under reduced pressure, yielding curcumin-loaded CaCO_3_. The obtained powder was further dried overnight in a desiccator to remove residual solvent.

This procedure follows the general solvent evaporation approach reported in previous studies.^24,25^

### Characterization of the Synthesized Materials

X-ray diffraction (XRD, Multiflex-120NP, Rigaku, Japan) was conducted using CuKα radiation (λ = 0.154 nm) over the 2θ range of 10°–70° at a scanning speed of 5°/min, and the patterns obtained were analyzed using MDI JADE 6.0. The specific surface areas of the ACC samples were determined from their N_2_ gas adsorption isotherms using the multipoint Brunauer–Emmett–Teller (BET, Autosorb-1-c/MK2, Quantachrome, USA) method. The Average pore size and pore volume was determined by the BJH method. Prior to the measurements, the samples were degassed for 5 h at 100 °C under vacuum conditions to remove adsorbed solvent molecules. The surface morphology of the ACC particles was evaluated using scanning electron microscopy (SEM, JSM-6380A, JEOL, Japan) at ×10,000 magnification. Simultaneous thermogravimetry and differential thermal analysis (TG-DTA, Exstar 6200N, Seiko Instruments, Japan) was performed over the temperature range of 20–600 °C at a heating rate of 10 °C/min under compressed air.

### Curcumin Release Study

Dissolution tests were performed as follows. ACC samples loaded with 30 μg/mL curcumin were dissolved in distilled water and shaken at 37 °C in a constant-temperature bath (ML-10F, Taitec Corporation, Japan) at 100 rpm. Sampling was conducted by withdrawing 1 mL aliquots of the solutions at 2, 5, 10, 30, 60, 90, and 120 min. The samples were filtered through a 0.22 μm filter and then analyzed by high-performance liquid chromatography (HPLC, SPD-20AD, Shimadzu, Japan). The HPLC conditions were as follows: column = COSMOSIL 5C18-MS-II (5 μm, 4.6 × 50 mm, Nacalai Tesque, Japan), column temperature = 40 °C, mobile phase = acetonitrile +0.1% phosphoric acid (45:55, v/v), flow rate = 1.0 mL/min, and injection volume = 10 μL.

## Results and Discussion

### Physical Evaluation of the CaCO_3_ Samples

XRD analysis was conducted to evaluate the crystalline structures of ACC samples prepared with citric acid. Figure 1a shows the XRD patterns of ACC samples prepared with 0.00–0.33% w/v citric acid. Peaks corresponding to both vaterite and calcite are clearly observed. However, when 0.33% w/v citric acid was added to the ACC dispersion, the peaks corresponding to vaterite disappeared and only one peak corresponding to calcite (29.48°) remained. This result may be attributed to the dissolution of vaterite at low pH and is supported by the results of a previous study.^4^

**Figure 1:**
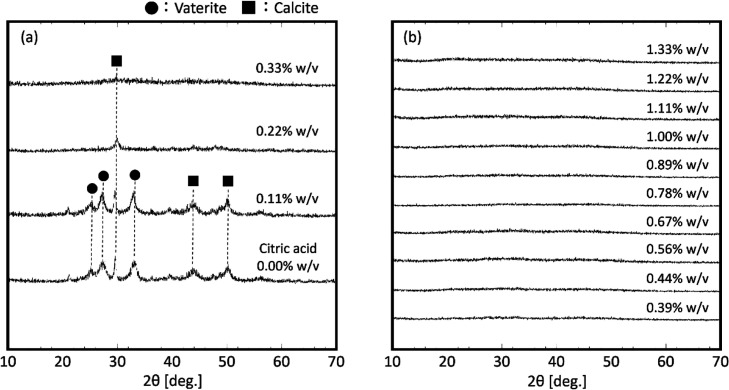
XRD patterns of ACC samples prepared with (a) 0.00–0.33 and (b) 0.39–1.33% w/v citric acid.

The pH values of the ACC dispersion after the addition of citric acid were experimentally measured, and the results are summarized as follows: 0 g: 8.52, 0.1 g: 10.55, 0.2 g: 10.70, and 0.3 g: 10.73. It should be noted that these measurements were performed in a mixed ethanol/ethylene glycol system containing limited water. Therefore, the reported values represent apparent pH rather than true aqueous pH and should be interpreted as indicative of the relative basicity of the system. Despite the addition of citric acid, the apparent pH remains in an alkaline range, which is attributed to the buffering effect of calcium carbonate and the nonaqueous solvent environment. Accordingly, caution is required when directly correlating these values with conventional aqueous pH-dependent behavior.

Specifically, Febrida et al. (2021) synthesized vaterite in basic environments and noted both the disappearance of the XRD peaks of vaterite as well as a change in its crystalline structure under acidic pH. The lack of significant peaks in the XRD patterns of the prepared materials may be attributed to their disordered state.^26^

Figure 1b shows the XRD patterns of ACC samples prepared with 0.39–1.44% w/v citric acid. The complete disappearance of the characteristic peaks for vaterite and calcite indicates the dissolution of these polymorphs under acidic conditions. A comparison of the XRD patterns of all the samples suggests the polymorphic transformation of CaCO_3_. The degree of crystallinity of the sample particles can also be affected by the concentration of the additive. In this study, the crystallinity of the ACC samples decreased as the amount of citric acid increased.

SEM was performed to examine the morphological properties of the ACC samples prepared with different amounts of citric acid, as shown in Figure 2. The dumbbell-shaped and spherical particles shown in Figure 2a–d confirmed that the particles produced were vaterite and calcite.^21^ The average particle diameter was 200 nm (Figure 2a–c); with the addition of 0.11% w/v citric acid, however, the average particle diameter doubled to 400 nm. Irregularly shaped particles were formed when 0.44–1.33% w/v citric acid was added to the ACC dispersion (Figure 2e–h). The particles aggregated, and the average particle diameter was less than 50 nm. Aggregated particles measuring 1 μm in size can also be observed. Previous research revealed irregularly shaped ACC particles that aggregated to form single particles with a size of 50 nm.^27^ The synthesized particles (Figure 2e–h) were presumed to be ACC because the XRD patterns in Figure 1b did not show peaks for the materials prepared over this range of citric acid concentrations owing to the amorphous nature of ACC. It should be noted that the SEM observations in this study were primarily intended to capture overall morphological trends as a function of citric acid concentration, rather than to resolve detailed primary particle structures. Therefore, the magnification used here is considered sufficient for the purpose of discussing the relationship between morphology and macroscopic properties.

**Figure 2:**
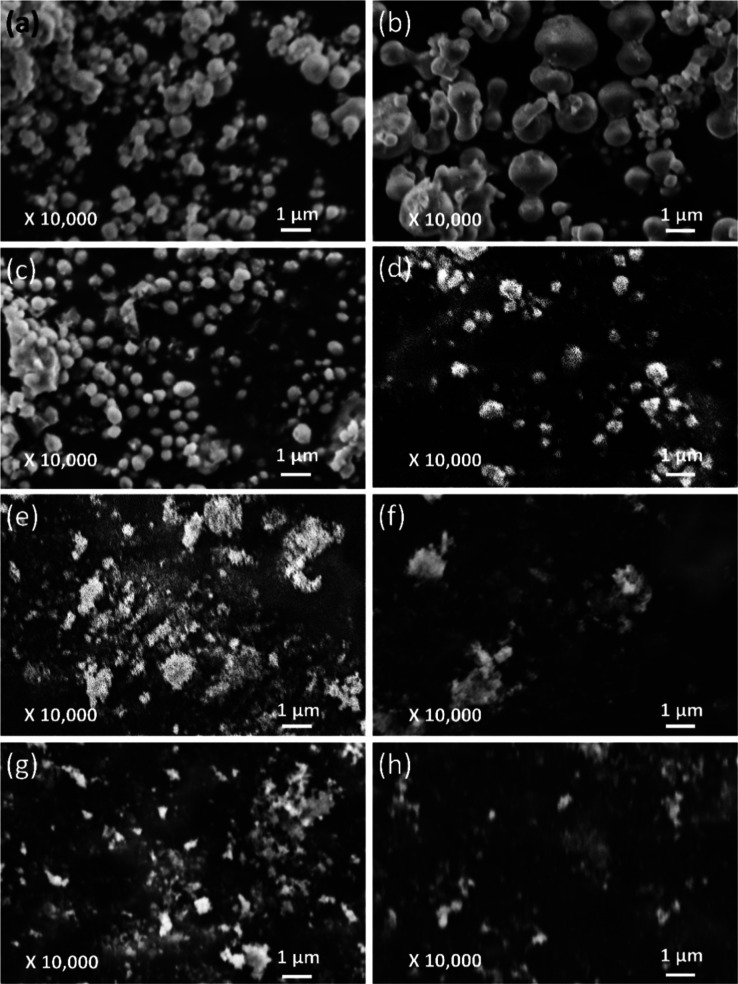
SEM images of ACC particles prepared with (a) 0.00, (b) 0.11, (c) 0.22, (d) 0.33, (e) 0.44, (f) 0.89, (g) 1.11, and (h) 1.33% w/v citric acid.

Figure 3 shows the specific surface areas calculated for ACC samples synthesized with different amounts of citric acid. The specific surface area of the ACC particles increased as the amount of citric acid added to the ACC dispersions increased from 0.00 to 0.39% w/v, reached equilibrium between 0.39 and 1.11% w/v, and then decreased with additions of over 1.11% w/v. When 0.39–1.11% w/v citric acid was added to the dispersions, the resulting ACC particles exhibited the highest specific surface areas, ranging from 600 to 700 m^2^/g. These values are comparable to those reported by Sun et al.,^28^ who obtained a specific surface area of 644 m^2^/g for ACC prepared using citric acid. Citric acid effectively penetrated and interacted with the surfaces of the ACC particles, contributing to the stabilization of ACC with a higher surface area. However, as more acid was added to the dispersions, the surfaces of the resulting particles “flattened” and their specific surface area decreased. This finding is supported by the results of Tang et al.,^29^ who showed that the pore size of coal particles increased with increasing pH, reflecting a decrease in specific surface area with the addition of an acid.

**Figure 3:**
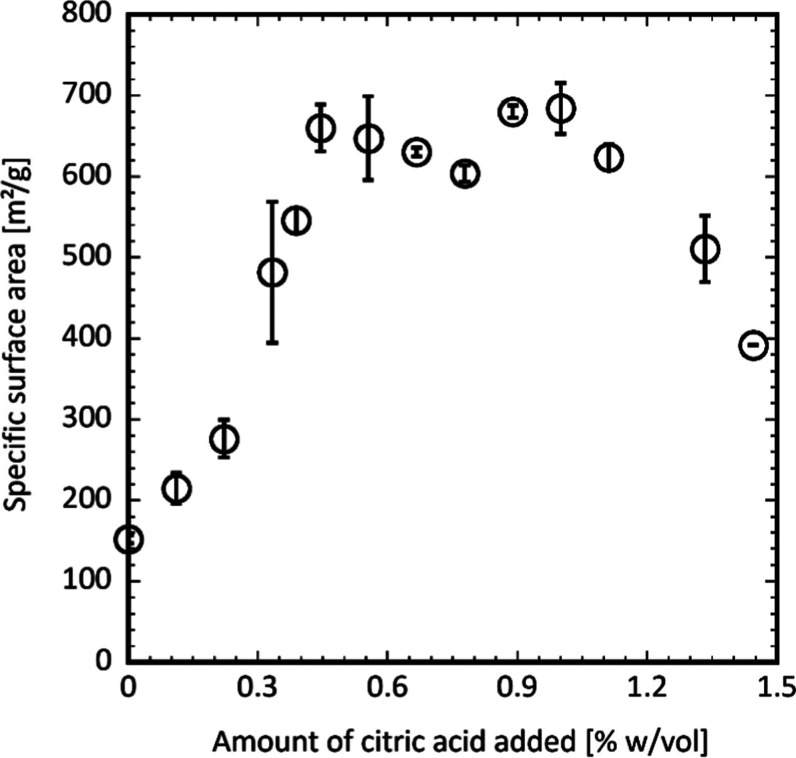
Specific surface area of ACC samples synthesized with different citric acid amounts.

Figure 4a shows the typical N_2_ adsorption–desorption isotherm of the representative ACC sample, exhibiting characteristics of a mesoporous structure. The corresponding pore size distribution (Figure 4b) confirms that the dominant pore sizes are larger than the molecular dimensions of curcumin. Additional pore size and pore volume analysis (see Table 1) indicates that the minimum average pore diameter (3.6 nm) exceeds the molecular dimensions of curcumin (∼2 nm), suggesting that molecular diffusion is not sterically hindered in the present system. Under these conditions, the specific surface area rather than pore size is considered the dominant factor governing curcumin dissolution behavior. This interpretation is consistent with previous reports on high-surface-area porous carriers, in which dissolution enhancement reaches a plateau beyond a threshold surface-area-to-cargo ratio.

**Figure 4:**
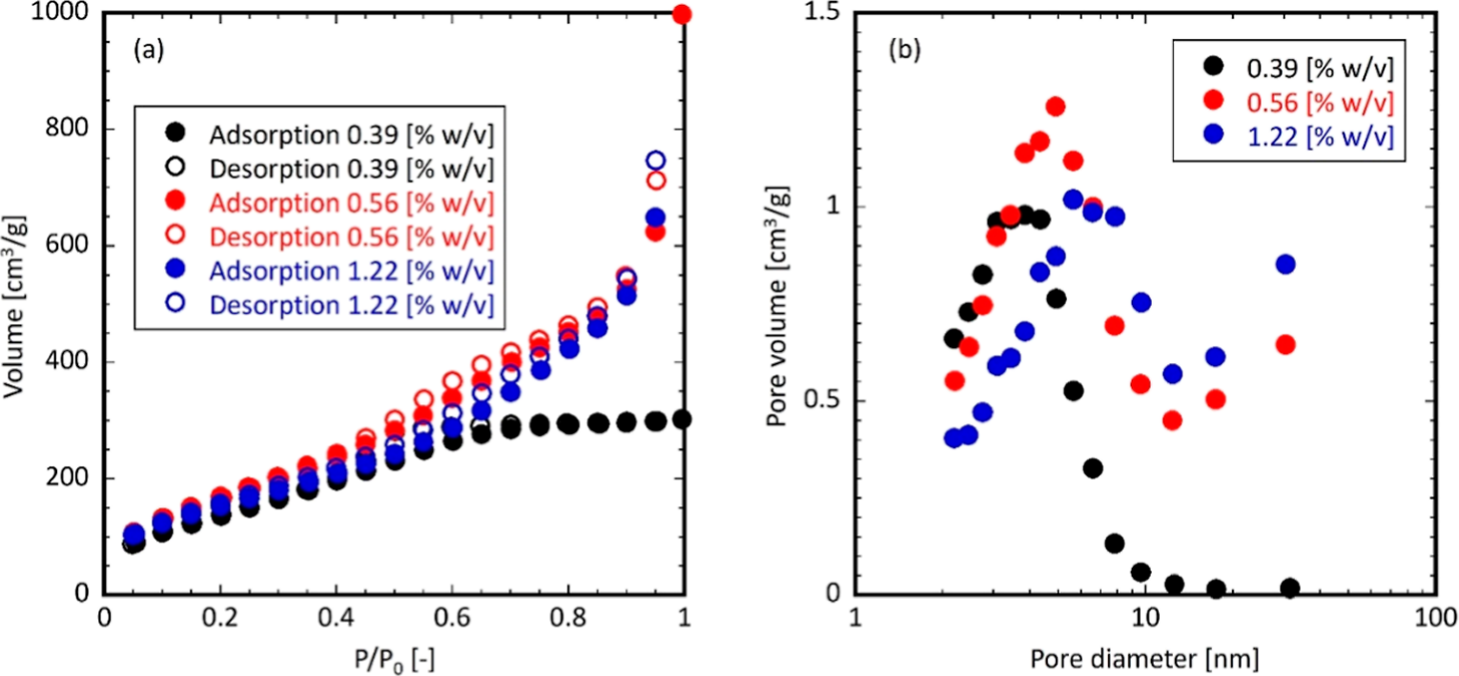
(a) N_2_ adsorption–desorption isotherms and (b) pore size distribution of a representative ACC sample.

**Table 1:** Average Pore Diameter and Pore Volume of a Representative ACC Sample Synthesized with Different Citric Acid Amounts

amount of citric acid added [% w/v]	average pore diameter [nm]	pore volume [cm^3^/g]
0.39	3.6	0.47
0.56	9.6	1.55
1.22	15.1	2.17

At higher citric acid concentrations, the observed increase in average pore diameter is considered to result from partial dissolution of ACC followed by reprecipitation. In this process, excessive acid promotes localized dissolution of the ACC structure, and subsequent structural rearrangement leads to coarsening of the pore network. Such a dissolution–reprecipitation mechanism can account for the simultaneous increase in pore size and decrease in specific surface area.

The synthesized samples were subjected to TG-DTA to evaluate the relationship between their specific surface area and citric acid addition. Here, citric acid content was calculated based on their weight loss rate. According to the TG-DTA results, significant weight loss occurred between 180 and 200 °C, corresponding to the onset of citric acid vaporization, and continued until approximately 300 °C. This trend is consistent with the findings of,^30^ who reported that the removal of water and CaCO_3_-related components occurs at temperatures of up to 200 °C, while the decomposition of organic matter occurs at temperatures of up to 500 °C.

Figure 5 compares the actual amounts of citric acid added to the ACC samples with those determined from the TG/DTA calculations. The calculated citric acid content in the samples increased as the amount of citric acid added to the ACC dispersions increased. However, the actual citric acid content increased to 0.22% w/v and reached equilibrium after 0.22% w/v. Differences in specific surface area may explain why the actual citric acid content reached equilibrium earlier than the calculated citric acid content. The ACC samples prepared with 0.44–1.11% w/v citric acid contained 10.5–14.7% citric acid, which means only a portion of the citric acid was adsorbed on the surface of the particles. The unadsorbed citric acid was likely removed during ultrasound-assisted ethanol washing after the aging process.^31,32^

**Figure 5:**
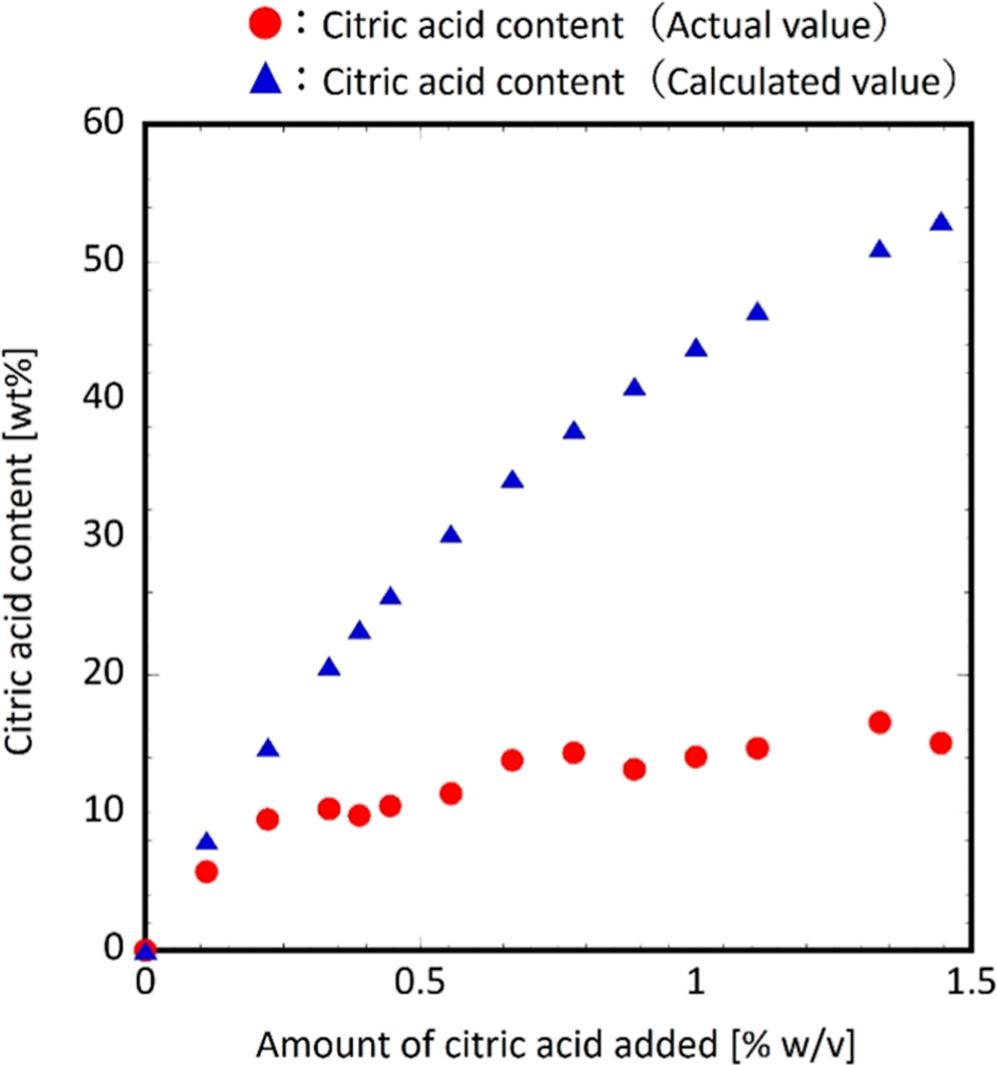
Comparison of actual and calculated citric acid contents in different ACC samples.

### Mechanism of Particle Growth and Crystallization in the Presence of Citric Acid

The mechanism of the particle growth and crystallization of ACC without citric acid addition is shown in Figure 6a. ACC has a porous structure (empirical formula: CaCO_3_·*n*H_2_O), with water molecules randomly surrounding the Ca^2+^ and CO_3_^2–^ ions. The phase transition of CaCO_3_ to its crystalline polymorphs, such as vaterite and calcite, is caused by the dissolution and recrystallization of ACC.^21,33^ ACC is thermodynamically unstable and dissolves in trace amounts of water and ethylene glycol. Particle growth and the partial dehydration of bound water occur as supersaturated ACC recrystallization repeatedly occurs until all of the water is completely removed. Complete dehydration causes ACC to crystallize and aggregate into crystalline polymorphs such as calcite and vaterite.

**Figure 6:**
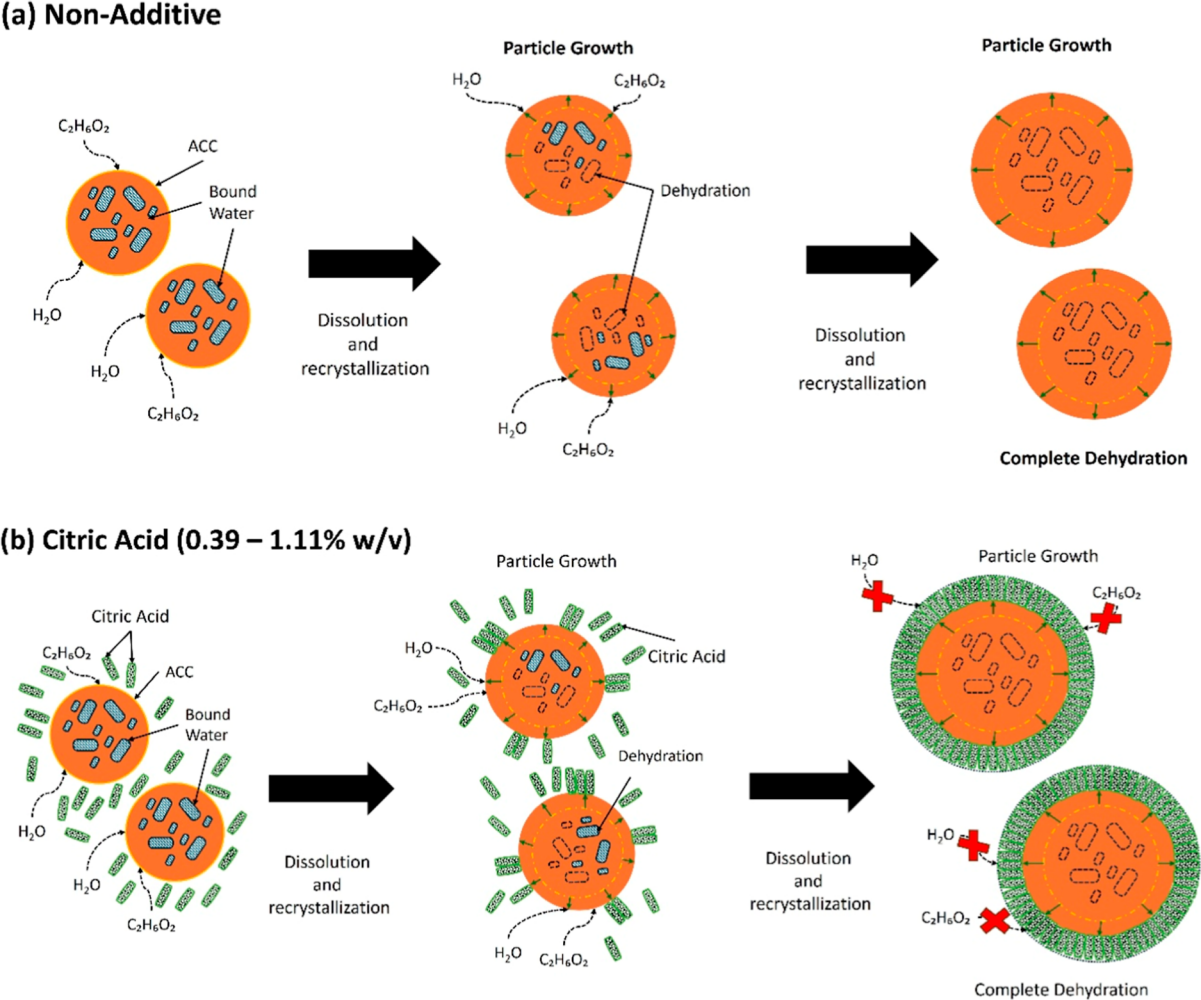
Mechanisms of the particle growth and crystallization of ACC particles (a) without and (b) with citric acid. This schematic illustrates a hypothesized mechanism based on literature reports, and does not represent direct experimental evidence.

The mechanism of the particle growth and crystallization of ACC with citric acid addition.

(0.39–1.11% w/v) is shown in Figure 6b. The stabilization of ACC observed in this study is not directly demonstrated at the molecular level, but is interpreted as being consistent with previously reported citrate–calcium carbonate interactions. Previous studies have shown that citrate species can interact with CaCO_3_-related phases through multidentate carboxylate coordination, hydrogen bonding, and electrostatic interactions, which suppress dissolution–recrystallization processes and delay polymorphic transformation.^14,16,34,35^ Accordingly, the inhibition of crystallization observed at appropriate citrate concentrations in the present system is considered compatible with these reported mechanisms, rather than being a direct spectroscopic verification.

In this study, the term “stabilization” is primarily defined as the suppression of rapid crystallization of ACC during the synthesis and curcumin-loading processes. To further evaluate the stability of the dried ACC, a representative sample prepared with 1.22% w/v citric acid was subjected to storage under controlled conditions (20 °C and 50% relative humidity), and its structural evolution was monitored by XRD over a period of 0–7 days (see Figure S1). No crystalline peaks corresponding to vaterite or calcite were detected at any time point, indicating that the amorphous structure was retained throughout the tested period. These results suggest that citrate-stabilized ACC maintains its amorphous state for at least several days after drying under moderate humidity conditions, which is relevant to typical handling and formulation processes.

It should be noted, however, that a comprehensive evaluation of long-term stability, including detailed humidity dependence and extended storage periods, is beyond the scope of the present study. Therefore, the present results are presented as a representative example of short-term stability under controlled conditions.

To provide more direct evidence for the interaction between citric acid and ACC, FT-IR measurements were performed for a representative sample prepared with 1.22% w/v citric acid. The FT-IR spectrum (see Figure S2) shows the disappearance of the characteristic C=O stretching band of protonated carboxylic acid near 1700 cm^–1^, accompanied by the emergence of a band at approximately 1580 cm^–1^, which is assignable to the asymmetric stretching vibration of carboxylate (COO^–^) groups. This spectral change indicates that citric acid exists predominantly in a deprotonated form on the ACC surface, providing spectroscopic evidence of its interaction with Ca-containing species. In addition, a new band observed at approximately 620 cm^–1^ is attributed to low-frequency Ca–O vibrational modes distinct from those of crystalline calcium carbonate, suggesting the presence of calcium–citrate-related interactions.

These spectral features are consistent with previously reported carboxylate coordination behavior in citrate–CaCO_3_ systems,^16^ where multidentate binding of citrate species to calcium ions has been proposed to suppress dissolution–recrystallization processes and stabilize amorphous phases.

Although these results provide spectroscopic indications of citrate–ACC interactions, a definitive determination of the coordination structure is beyond the scope of the present study. Accordingly, the observed stabilization is interpreted as being consistent with literature-reported mechanisms rather than as direct molecular-level confirmation.

When added at low levels (0.00–0.33% w/v), citric acid is unable to coat the ACC particle surfaces completely, promoting the dissolution of ACC in the solvent and its recrystallization to vaterite and calcite. When added at appropriate levels (0.39–1.11% w/v), citric acid can completely coat the ACC particle surfaces and inhibit the dissolution and recrystallization of ACC to vaterite and calcite. However, excess citric acid (1.11–1.33% w/v) reduces the specific surface area of ACC by promoting its dissolution, which leads to the enlargement of pores. Repeated cycles of ACC dissolution and recrystallization result in ACC particles with a smaller specific surface area.

### Surface Area Threshold for Curcumin Release from ACC

Regarding the stability of ACC during curcumin loading, three aspects should be considered. First, the inherent stability of the ACC template under low-water conditions has been established in our previous studies.^24^ Second, curcumin is maintained in a highly dispersed or amorphous state within the ACC matrix, which is advantageous for dissolution but may be sensitive to high humidity; accordingly, controlled storage conditions are required for practical use. Third, the chemical compatibility between CaCO_3_ and curcumin has been well documented, with no reports of chemical degradation during formulation.^36,37^ Taken together, while long-term stability under all conditions is beyond the scope of this study, the carrier–guest system is considered sufficiently stable within the time frame relevant to preparation and application.

The dissolution profiles of curcumin loaded on different CaCO_3_ samples are shown in Figure 7. To further clarify the relationship between specific surface area and dissolution behavior, the corresponding data for all samples are summarized in Table S1.

**Figure 7:**
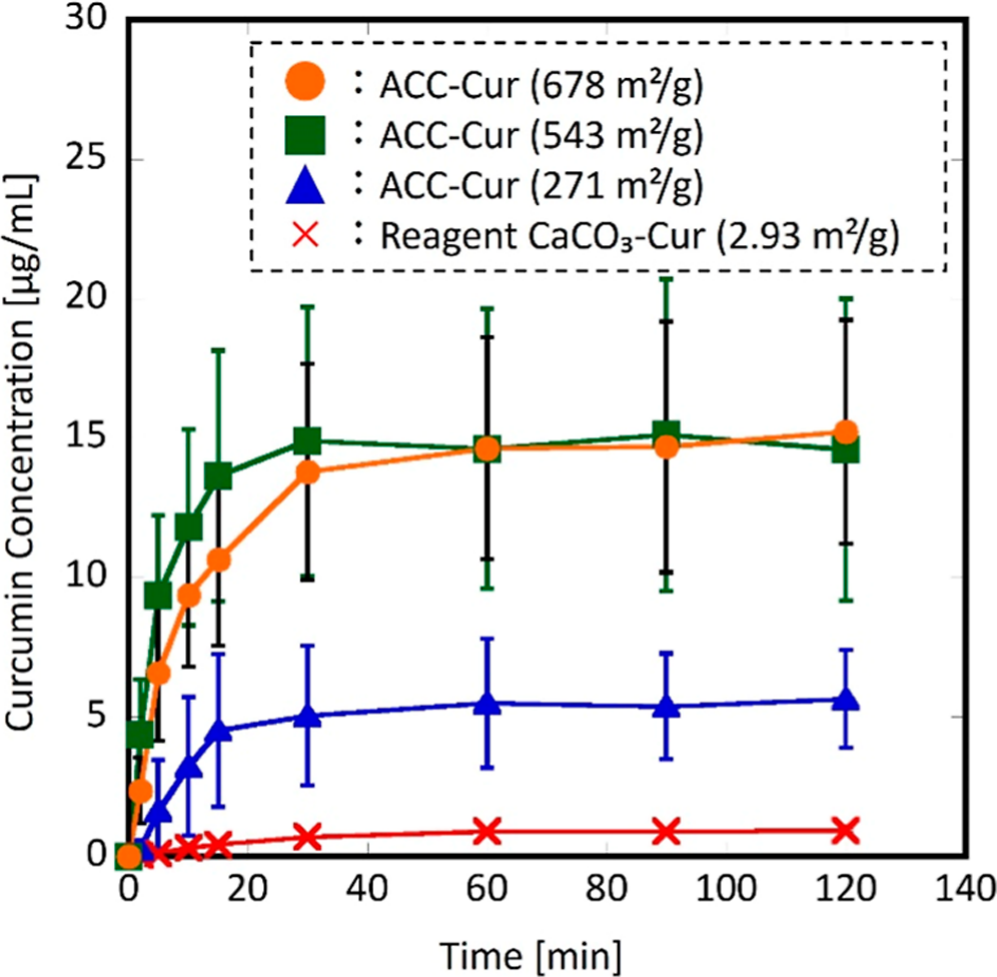
Comparison of curcumin release rates from ACC samples with varying surface areas.

In this study, curcumin was loaded onto ACC at a fixed initial ratio of 300 mg/g using a solvent evaporation method. For the dissolution experiments, the curcumin-loaded samples were dispersed in water at a concentration corresponding to 30 μg/mL of curcumin. Under these conditions, only a fraction of the loaded curcumin contributes to the measured dissolution behavior. Therefore, differences in dissolution profiles are primarily attributed to variations in the structural properties of the carrier, particularly the specific surface area, rather than differences in loading amount. As shown in Table S1, the dissolution of curcumin increases with increasing specific surface area but reaches a plateau above a certain threshold. This result supports the surface-area-dependent dissolution behavior discussed in this study.

After 120 min, the amounts of curcumin released from ACC-Cur0.56 (678 m^2^/g) and ACC-Cur0.39 (543 m^2^/g) reached approximately 15 μg/mL, corresponding to a dissolution rate of approximately 50%. By contrast, ACC-Cur0.22 (271 m^2^/g) and reagent CaCO_3_–Cur showed dissolution rates of only 17% and 3%, respectively. These results clearly indicate that CaCO_3_ carriers with a larger specific surface area are capable of releasing higher amounts of curcumin into the solution. This enhancement is attributed to the adsorption of curcumin onto the CaCO_3_ surface in a predominantly amorphous state. A larger specific surface area provides more adsorption sites, enabling the stabilization of curcumin in a highly dispersed, amorphous form, which encourages dissolution. Similar solubility-enhancement mechanisms have been widely reported for porous silica and other high-surface-area carriers, which promote drug dissolution by maintaining the guest molecules in molecularly dispersed or amorphous states.^38^ To place the present ACC-based system in context, it is useful to compare it with representative high-surface-area carriers that have been widely investigated for curcumin delivery, such as mesoporous silica, activated carbon, and porous starch. Mesoporous silica and activated carbon often exhibit high loading capacities and strong adsorption affinity for hydrophobic compounds; however, their application in functional foods is frequently limited by regulatory considerations, safety issues, or sensory constraints.^39–41^ Porous starch has attracted attention as a food-grade alternative, but it generally lacks intrinsic pH-responsive release behavior.^42,43^ In contrast, CaCO_3_ is an approved food additive and exhibits acid-responsive dissolution in gastric environments, which provides an additional functional advantage for oral delivery systems.^44^ Accordingly, the present study does not aim to replace existing carriers in all applications, but rather to demonstrate that citric acid–stabilized ACC represents a complementary, food-compatible platform with tunable structure-performance relationships.

Although local pH variations in the vicinity of dissolving ACC particles were not directly measured in this study, their influence on the observed dissolution behavior is considered to be limited under the present conditions. The dissolution of calcium carbonate in water is generally buffered by the carbonate–bicarbonate equilibrium system, resulting in a mildly alkaline bulk pH (typically pH 8–9). In addition, the low solid-to-liquid ratio and continuous agitation employed in this study are expected to suppress local pH gradients and promote rapid homogenization. Curcumin exhibits low solubility over a wide pH range and undergoes rapid degradation only at strongly alkaline conditions (pH > 10). Therefore, within the expected pH range of this system, the qualitative trends in dissolution behavior are unlikely to be significantly affected by pH variations.

Interestingly, no significant difference in dissolution rate was observed between ACC-Cur samples with surface areas of 678 and 543 m^2^/g. This result suggests that although high-surface-area ACC can effectively improve the solubility of curcumin, this enhancement plateaus once a certain surface area threshold is reached. Beyond this threshold, increases in surface area do not translate into further improvements in dissolution.^45^ These findings imply that future formulation strategies should focus on optimizing the surface-area-to-cargo ratio rather than simply maximizing the surface area of the carrier. From a practical formulation perspective, excessively rapid release of curcumin is undesirable because curcumin is chemically unstable under acidic gastric conditions.^46,47^ Controlled release behavior, combined with amorphization-induced supersaturation (the so-called “spring effect”), is therefore more effective for improving oral bioavailability of poorly water-soluble compounds.^48^ Moreover, high curcumin loading capacity enables delivery of practical daily doses in compact dosage forms, thereby fulfilling key requirements for functional foods and nutraceutical products.

Taken together, the results collectively reveal that the ability to tune the polymorphism and surface characteristics of ACC through citric acid-mediated synthesis provides a promising platform for designing functional food and pharmaceutical formulations with controlled-release properties. In this study, the term “stabilization” is used to describe the suppression of rapid crystallization of ACC during the material preparation and curcumin-loading processes, rather than long-term thermodynamic stability. This definition reflects the experimental time frame relevant to synthesis, handling, and formulation, and does not imply indefinite structural persistence over extended storage periods or under all environmental conditions.

## Conclusion

ACC particles were successfully synthesized under ambient conditions using different citric acid concentrations to enable the precise control of particle growth and stabilization. XRD analysis confirmed that addition of 0.39–1.11% w/v citric acid completely suppressed ACC crystallization. SEM observations supported this finding, revealing irregularly shaped aggregated particles that are characteristic of stabilized ACC. The suppression of crystallization observed in this study is consistent with previously reported interactions between citrate species and calcium carbonate phases, although direct molecular-level evidence was not obtained here. Thus, ACC maintains an amorphous structure with a significantly enlarged specific surface area. Collectively, these observations identify a reproducible citrate stabilization window that yields amorphous ACC with surface areas exceeding 500–700 m^2^/g, providing practical processing criteria for preparing application-ready high-surface-area carriers.

The functional performance of the prepared ACC particles was confirmed by curcumin dissolution studies. High-surface-area ACC (678 and 543 m^2^/g) released approximately 50% of the loaded curcumin within 120 min, whereas low-surface-area ACC (271 m^2^/g) and reagent crystalline CaCO_3_ showed curcumin dissolution rates of only 17% and 3%, respectively. These results demonstrate that increasing the specific surface area of ACC markedly enhances curcumin release by maintaining the compound in a highly dispersed amorphous state on the ACC surface. Notably, the dissolution profiles of ACC samples with specific surface areas of 678 and 543 m^2^/g were nearly identical, suggesting that solubility enhancement plateaus once a certain surface area threshold is achieved. This plateau behavior highlights a practical design rule: optimizing—not merely maximizing—the surface-area-to-cargo ratio is key to engineering effective ACC-based delivery systems. This principle is broadly applicable to other poorly soluble nutraceuticals and hydrophobic bioactives.

In summary, this study demonstrated that the citric acid-mediated synthesis of ACC is a precise strategy for preparing stable, high-surface-area ACC samples with tunable structural properties. These biocompatible materials show strong potential as carriers for functional food ingredients and bioactive compounds, offering improved dissolution behavior and opportunities for controlled-release formulation designs. The ability to tailor the polymorphs and surface characteristics of ACC opens new avenues for the development of advanced nutraceutical delivery systems.

## Supplementary Material



## Notes

Details of short-term stability evaluation (Figure S1); FT-IR analysis of citrate–ACC interaction (Figure S2); and relationship between specific surface area and curcumin dissolution behavior (Table S1) (PDF)

## ∥

Department of Chemical and Environmental Engineering, Malaysia-Japan International Institute of Technology (MJIIT), Universiti Teknologi Malaysia Kuala Lumpur, Jalan Sultan Yahya Petra, Kuala Lumpur 54100, Malaysia
