# Supplementary material for: Title: Citric Acid-Stabilized Amorphous CaCO3 as a High-Surface-Area Carrier for Functional Food Ingredients
Source: ACS Omega. 2026 Jul 20;11(30):44903–12. doi: 10.1021/acsomega.6c02212 (PMC13448995; doi:10.1021/acsomega.6c02212)
Supplement: Supplementary file 1 [file ao6c02212_si_001.pdf]

Supporting Information

Citric acid-stabilized amorphous $CaCO_3$ as a high-surface-area carrier for functional food ingredients

Daichi Sakawaki[1], Khairunnisa Mohd Paad[1,#], Rena Nagaoka[1], Yasuteru Mawatari[1], Mayumi Ikeda-Imafuku[2], Tatsuya Fukuta[2], Prakasan Nisha[3], Kazunori Kadota[2], Ayaka Sawada[1], Shinya Yamanaka[1,*]

[1] Division of Applied Sciences, Muroran Institute of Technology, Mizumoto-cho 27-1, Muroran 050-8585, Japan

[2] Department of Physical Pharmaceutics, School of Pharmaceutical Sciences, Wakayama Medical University, 25-1 Shichibancho, Wakayama 640-8156, Japan

[3] Agro and Food Processing Technology Division, CSIR-National Institute for Interdisciplinary Science and Technology (CSIR-NIIST), Thiruvananthapuram 695019, Kerala, India.

# present address

Department of Chemical and Environmental Engineering, Malaysia-Japan International Institute of Technology (MJIIT), Universiti Teknologi Malaysia Kuala Lumpur, Jalan Sultan Yahya Petra, Kuala Lumpur 54100, Malaysia

Corresponding author:

*Shinya Yamanaka (**syama@muroran-it.ac.jp**)

Division of Applied Sciences, Muroran Institute of Technology, Mizumoto-cho 27-1, Muroran 050-8585, Japan

Short-term Stability of Dried ACC

The short-term stability of the dried ACC was evaluated under controlled environmental conditions. The ACC sample prepared with 1.22% w/v citric acid was stored at 20 °C and 50% relative humidity. The structural evolution was monitored by X-ray diffraction (XRD) at 0, 1, 3, and 7 days using a diffractometer (Multiflex-120NP, Rigaku) with CuKα radiation (λ = 0.154 nm) in the 2θ range of 10–70°.

Figure S1 shows the XRD patterns of the stored samples. No diffraction peaks corresponding to crystalline phases such as vaterite or calcite were observed throughout the storage period, indicating that the amorphous structure of ACC was retained for at least 7 days under the tested conditions.

These results demonstrate that the citrate-stabilized ACC exhibits sufficient short-term stability for handling and formulation processes under moderate humidity conditions.

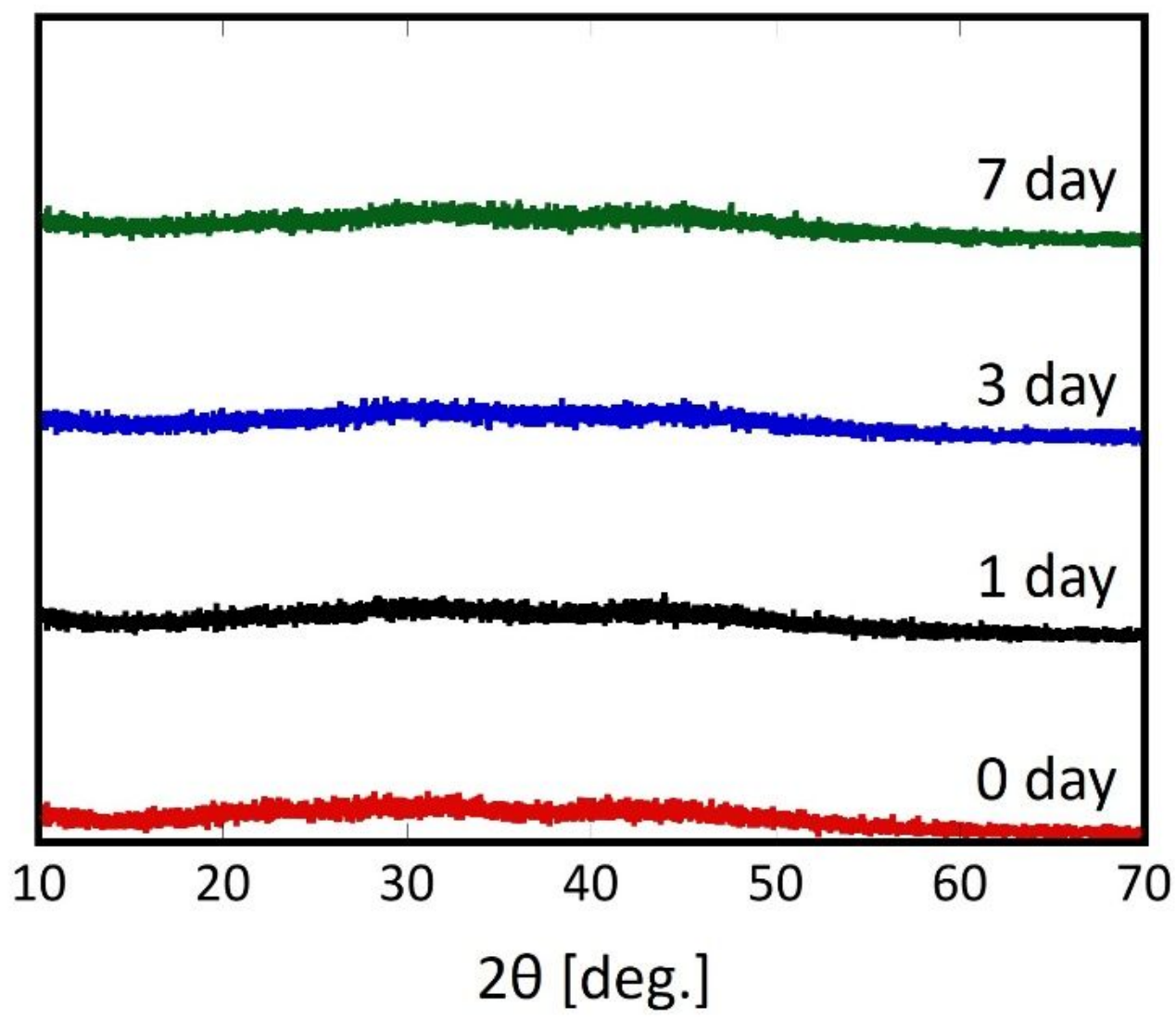

Figure S1. XRD patterns of citrate-stabilized ACC (1.22% w/v) after storage at 20 °C and 50% RH for 0–7 days. No crystalline peaks are observed, indicating that the amorphous structure is maintained during the storage period.

FT-IR analyses

FT-IR measurements were conducted to examine the interaction between citric acid and ACC. The spectra of powder samples were recorded directly on a Shimadzu IRSpirit FT-IR spectrometer equipped with a QATR-S single-reflection ATR accessory using a diamond prism, with a resolution of 4 cm^{-1} over the range of 4000–400 cm^{-1}. **Figure S2** shows the FT-IR spectra of ACC sample prepared with citric acid (1.22% w/v).

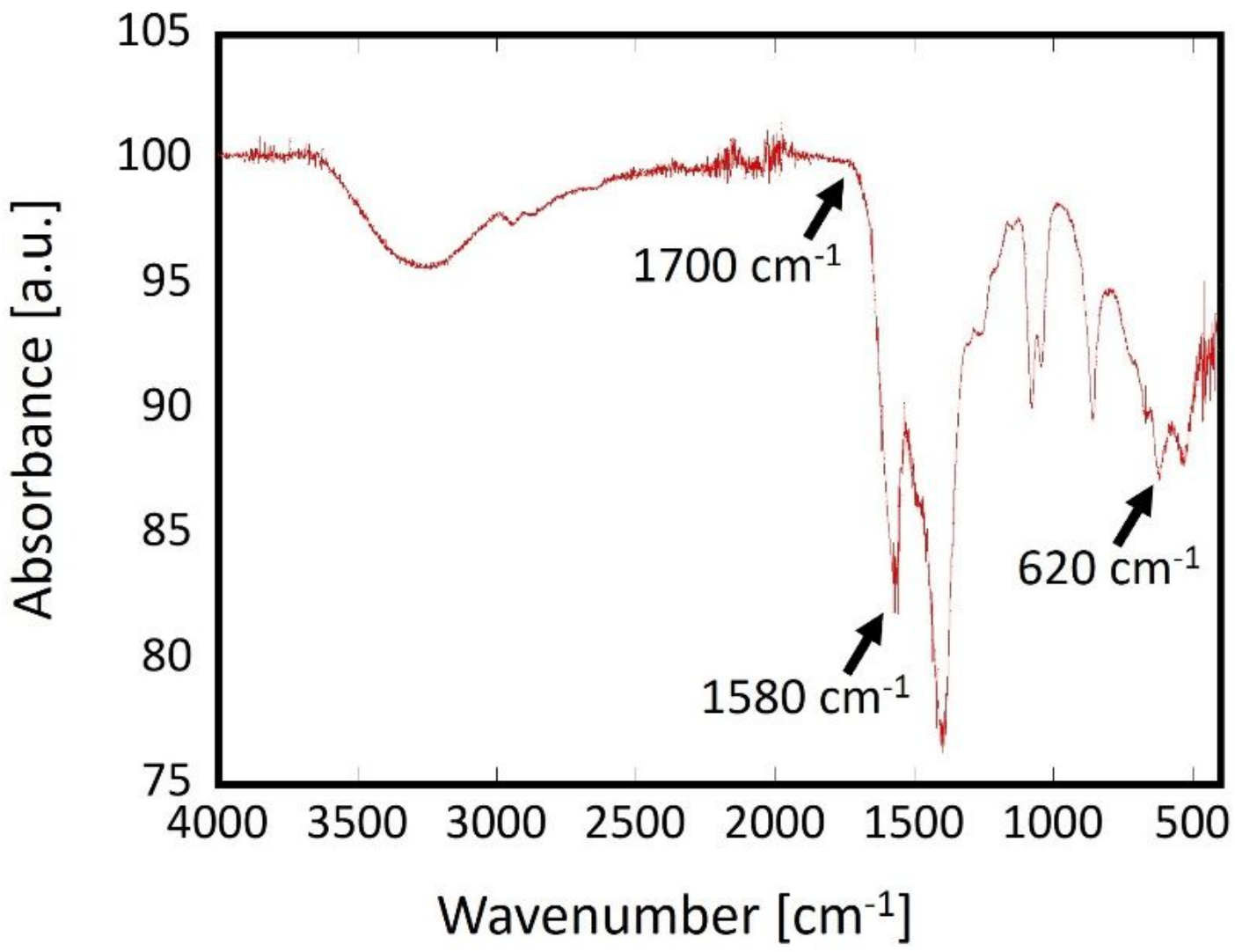

Figure S2. FT-IR spectra of ACC samples without citric acid and with 1.22% w/v citric acid. The disappearance of the C=O band (~1700 cm^{-1}) and the appearance of the COO^{-} band (~1580 cm^{-1}) indicate the formation of carboxylate species on the ACC surface.

Curcumin loading and release mapping

To clarify the relationship between specific surface area and dissolution behavior, the relevant data for all samples are summarized in **Table S1**.

In this study, curcumin was loaded onto ACC at a fixed initial ratio of 300 mg/g using a solvent evaporation method. For the dissolution experiments, curcumin-loaded samples were dispersed in water at a concentration corresponding to 30 µg/mL of curcumin.

Under these conditions, only a fraction of the loaded curcumin contributes to the measured dissolution behavior. Therefore, differences in dissolution profiles are primarily attributed to variations in the structural properties of the carrier, particularly specific surface area.

As shown in Table S1, the amount of curcumin released increases with increasing specific surface area, but reaches a plateau above a certain threshold. These results support the surface-area-dependent release behavior discussed in the main text.

Table S1. Relationship between specific surface area and curcumin dissolution behavior for ACC samples prepared with different citric acid concentrations.

Citric acid [% w/v]	SSA [m^2/g]	Release [μg/mL at 120min]
0	2.93	0.9
0.22	271	5.7
0.39	543	14.6
0.56	678	15.2

*Curcumin was loaded at a fixed initial ratio of 300 mg/g. Dissolution tests were performed at a concentration of 30 μg/mL, and the reported values correspond to the amount of curcumin released into solution after 120 min.
